# Early β-blocker use and in-hospital outcomes in patients with chronic obstructive pulmonary disease hospitalized with acute coronary syndrome: findings from the CCC-ACS project

**DOI:** 10.3389/fcvm.2024.1385943

**Published:** 2024-07-11

**Authors:** Tao Zhang, Xu Wang, Yucheng Zhang, Tingting Feng, Yujie Zhou, Lin Zhao

**Affiliations:** ^1^ Center for Coronary Artery Disease, Beijing Anzhen Hospital, Capital Medical University, Beijing, China; ^2^ Department of Cardiology, Beijing Anzhen Hospital, Capital Medical University, Beijing, China; ^3^ Emergency and Critical Care Center, Beijing Anzhen Hospital, Capital Medical University, Beijing Institute of Heart Lung and Blood Vessel Disease, Beijing, China

**Keywords:** β-blocker, chronic obstructive pulmonary disease, acute coronary artery syndrome, in-hospital outcomes, early use

## Abstract

**Background:**

Patients with chronic obstructive pulmonary disease (COPD) after acute coronary artery syndrome (ACS) are at an increased risk of heart failure and death. However, β-blockers have been underused in this population group due to concerns of adverse reactions.

**Objective:**

This study aims to investigate the β-blocker prescription at admission and its impact on the in-hospital outcomes in patients with COPD after ACS in a Chinese national cohort.

**Methods:**

Among 113,650 patients with ACS enrolled in the national registry of the Improving Care for Cardiovascular Disease in China between November 2014 and July 2019, a total of 1,084 ACS patients with COPD were included in this study. The primary endpoint was in-hospital mortality, and the secondary endpoint was the composite of in-hospital all-cause death and heart failure.

**Results:**

Early oral β-blocker therapy was administered to 49.8% of patients. The Kaplan–Meier analysis showed that the early β-blocker treatment group had lower all-cause mortality (0.9% vs. 2.9%; *P* < 0.05) and lower combined endpoint event rate (8.2% vs. 12.0%; *P* < 0.05) compared to the those of the non-early β-blocker treatment group. The analysis of inverse probability of treatment weighting showed that the early β-blocker treatment group was associated with a significantly reduced incidence of all-cause death (risk ratio, 0.332, 0.119–0.923, *P* = 0.035), heart failure (risk ratio, 0.625, 95% CI 0.414–0.943, *P* = 0.025), and combined endpoint events (risk ratio: 0.616, 95% CI: 0.418–0.908, *P* = 0.014). In the subgroup of patients over 70 years of age, the corresponding hazard ratio was 0.268 (95% CI 0.077–0.938) for all-cause mortality and 0.504 (95% CI 0.316–0.805) for combined endpoint events.

**Conclusion:**

β-blockers have been underused in patients with COPD and ACS in China. Early β-blocker therapy is associated with an improvement in in-hospital outcomes in patients with COPD after ACS.

**Clinical Trial Registration:**

ClinicalTrials.gov, identifier (NCT02306616).

## 1 Introduction

Patients with acute coronary syndrome (ACS) and chronic obstructive pulmonary disease (COPD) are a high-risk population. Compared to patients without COPD, patients with COPD after acute myocardial infarction (AMI) have a higher risk of heart failure (1) and mortality (2, 3). Hypoxia and systemic inflammation may be involved in the pathophysiological interactions between ACS and COPD (4). Thus, it is important to reduce adverse events after ACS in this population.

The inadequate use of revascularization and secondary prevention medications are potential reasons for the mortality gap in patients with COPD following AMI (2). β-blockers have been underused in patients with COPD and ACS due to concerns about their adverse effects on the respiratory function of patients with COPD (5). The clinical benefits of β-blockers after ACS have been proven by contemporary trials (6, 7). However, the evidence of early β-blocker use in COPD patients following ACS remains limited.

This study aims to explore the association between early β-blocker use (within 24 h of admission) and in-hospital outcomes in patients with COPD and ACS in a national registry and identify the factors influencing the use of β-blockers in patients with COPD.

## 2 Materials and methods

### 2.1 Participants

The Improving Care for Cardiovascular Disease in China-ACS Project (CCC-ACS), a collaborative effort by the American Heart Association and the Chinese Society of Cardiology, is an ongoing national quality improvement program launched in November 2014 and involves 150 tertiary hospitals in China. Since 2017, the CCC-ACS program has extended to 82 secondary hospitals and another 8 tertiary hospitals. Details of the study design were reported in a previous study (8). The CCC-ACS project was approved by the Institutional Review Committee of Beijing Anzhen Hospital, and informed consent was waived. This study was registered at ClinicalTrials.gov (NCT02306616) and is in accordance with the Helsinki Declaration. From November 2014 to July 2019, a total of 113,650 patients with ACS were enrolled in this study. We focused our analysis on participants with COPD.

### 2.2 Definitions of variables

The medical history and periprocedural details were obtained from the patients’ medical charts and entered into the database by trained data abstractors. Standardized definitions were utilized across all hospitals for variable collection. Since metoprolol and bisoprolol are the most commonly used β-blocker in Chinese patients with ACS, β-blocker users were defined as receiving these two kinds of β-blocker. Early oral β-blocker therapy was defined as the initiation of therapy within 24 h after admission. Non-early oral β-blocker therapies included oral β-blocker therapy initiated more than 24 h after admission or no β-blocker therapy administered during hospitalization. Patients who received early intravenous β-blocker were excluded from this analysis. The diagnoses of COPD were retrieved from the Improving Care for Cardiovascular Disease in China-ACS registry (CCC-ACS) using ICD-9 codes 491–492 and 496 and ICD-10 codes J41–J44, excluding cases coded for asthma. Myocardial infarction is defined as an increase of cardiac troponin with at least one value above the 99th percentile upper reference limit and ischemic symptoms and/or new or presumed new ST-segment, T-wave changes, or new left bundle branch block (9).

Patients meeting one of the following criteria were excluded: (1) cardiogenic shock or cardiac arrest at admission; (2) hemodynamic instability [systolic blood pressure (SBP) <85 mmHg or heart rate <50 beats/minute] at admission; (3) patients with acute heart failure at admission; (4) contraindications to β-blocker therapy, including second- or third-degree atrioventricular block and bradycardia; (5) mechanical complications (ventricular septal perforation, papillary muscle rupture, and myocardial rupture); (6) patients who received early intravenous β-blocker; and (7) length of hospital stay ≤1 day or >15 days.

### 2.3 Endpoints

The primary endpoint was in-hospital all-cause mortality. The secondary endpoint was combined endpoint events, including in-hospital all-cause death and heart failure.

### 2.4 Statistical analysis

Continuous variables with a normal distribution are presented as the means and standard deviations. Continuous variables with a skewed distribution are presented as the medians with 25th–75th percentiles. Differences in baseline characteristics were tested with the chi-square and *t*-test or Kruskal–Wallis tests for categorical and continuous variables, respectively. Kaplan–Meier methods were used to estimate the in-hospital event rates for each endpoint, and comparisons between the study groups were performed using the log-rank test.

To consolidate the findings, we also performed the inverse probability of treatment weighting (IPTW) using the propensity score method in the study cohort and compared the differences between the early β-blocker and non-early β-blocker treatment groups. Logistic regression was performed to estimate the propensity score (PS), getting the following variables adjusted: age, sex, current smoker, previous disease history [myocardial infarction (MI), percutaneous coronary intervention (PCI), diabetes, hypertension, ischemic stroke], Killip class, pre-hospital treatment in 2 weeks (β-blocker, ACEI/ARB, statin, aspirin), treatment within 24 h of admission (DAPT, P2Y12 inhibitor, aldosterone antagonist, ACEI/ARB, statin), and type of MI and PCI treatment. The IPTW was calculated by each individual based on his or her PS. Each case from the early β-blocker group was given a weight of Pt/PS, where Pt refers to the proportion of patients receiving early β-blocker among the whole cohort, and each case from the non-early β-blocker group was given a weight of (1-Pt)/(1-PS). In this way, we obtained a stabilized weight for each case of the study cohort, avoiding any extreme values that may result in unreliable outcomes.

All tests were two-sided with a *P*-value for significance of <0.05. All analyses were performed using SAS version 9.4 software (SAS Institute, Cary, NC, United States).

## 3 Result

### 3.1 Patient characteristics

**Figure 1:**
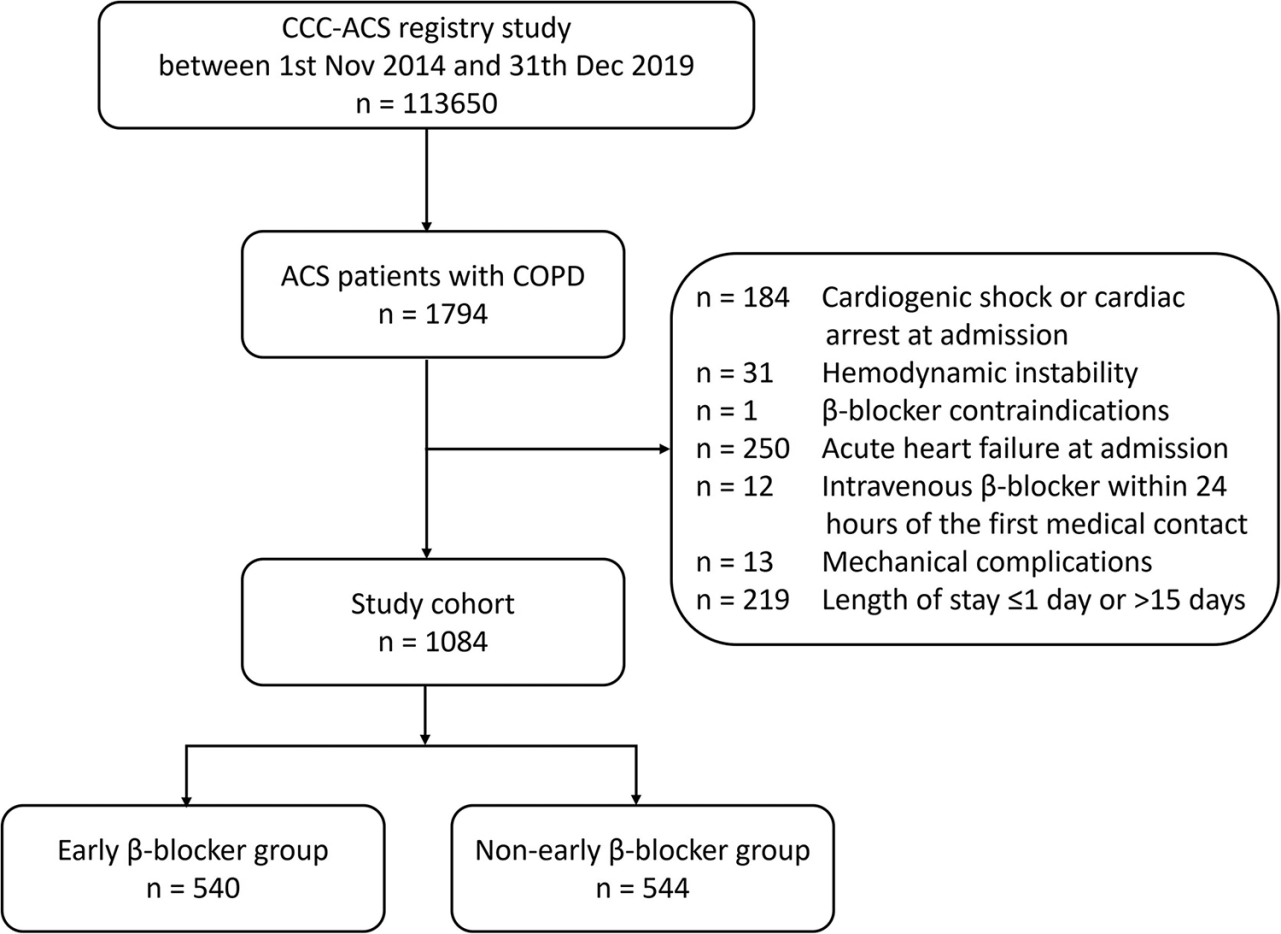
Flowchart of the study. ACS, acute coronary syndrome; CCC-ACS, Care for Cardiovascular Disease in China-Acute Coronary Syndromes; COPD, chronic obstructive pulmonary disease.

As shown in Figure 1, a total of 1,084 patients with COPD were identified in the CCC-ACS project with the inclusion and exclusion criteria applied. Among them, 540 (49.8%) patients had early oral β-blocker treatment within 24 h after admission. The baseline characteristics of the study cohort are summarized in Table 1. Compared to the non-early β-blocker treatment group, the early β-blocker treatment group had a significantly higher prevalence of hypertension (73.3 vs. 67.1%, *P* = 0.025). Cardiovascular medications were more frequently used at admission in the early β-blocker treatment group, including aspirin, angiotensin-converting enzyme inhibitor or angiotensin II receptor blocker, and statin (Table 2). After adjustment using the IPTW and propensity score-matched methods, the baseline characteristics were well-balanced (Supplementary Material Table S1).

**Table 1:** Characteristics of patients with ACS and COPD according to β-blocker use.

Variables	Early use of β-blocker (*n* = 540)	Non-early use of β-blocker (*n* = 544)	*P*-value
Age, year, mean (SD)	72.6 ± 9.2	73.0 ± 9.7	0.552
Male, *n* (%)	419 (77.6)	416 (76.5)	0.661
BMI, kg/m^2^, mean (SD)	23.5 ± 3.6	23.4 ± 4.1	0.867
Current smoker, *n* (%)	214 (39.6)	226 (41.5)	0.521
HBP, *n* (%)	396 (73.3)	365 (67.1)	0.025
DM, *n* (%)	191 (35.4)	193 (35.5)	0.970
Dyslipidemia, *n* (%)	59 (10.9)	52 (9.6)	0.458
STEMI at presentation, *n* (%)	248 (45.9)	261 (48.0)	0.499
Prior comorbidity, *n* (%)			
Heart failure	44 (8.1)	51 (9.4)	0.475
MI	66 (12.2)	50 (9.2)	0.107
Stroke	77 (14.3)	71 (13.1)	0.563
Peripheral arterial disease	27 (5.0)	23 (4.2)	0.545
Atrial fibrillation	36 (6.7)	30 (5.5)	0.428
History of myocardial revascularization, *n* (%)			
PCI	63 (11.7)	49 (9.0)	0.150
CABG	4 (0.7)	2 (0.4)	0.408
Blood pressure, mean (SD), mmHg			
SBP	132.4 ± 21.7	132.2 ± 21.8	0.857
DBP	77.8 ± 13.6	76.5 ± 12.8	0.112
Heart rate, beats/min, mean (SD)	80.7 ± 14.8	79.7 ± 17.6	0.331
Killip classesⅡ–Ⅲ, *n* (%)	211 (39.1)	258 (47.4)	0.006
eGFR, mean (SD), ml/min/1.73 m^2^	76.2 ± 21.2	75.3 ± 23.4	0.552
Renal insufficiency, *n* (%)	113 (20.9)	132 (24.3)	0.176
Serum creatinine level, mean (SD), mg/dl	1.0 ± 0.6	1.0 ± 0.5	0.788
Hemoglobin, g/L, mean (SD)	132.7 ± 20.4	131.1 ± 20.4	0.221
LVEF, mean (SD), %	55.4 ± 9.9	55.1 ± 9.9	0.651

BMI, body mass index; CABG, coronary artery bypass graft; DBP, diastolic blood pressure; DM, diabetes mellitus; eGFR, estimated glomerular filtration rate; HBP, high blood pressure; LVEF, left ventricular ejection fraction; MI, myocardial infarction; NA, not applicable; NSTEMI, non-ST-segment elevation myocardial infarction; PCI, percutaneous coronary intervention; SBP, systolic blood pressure; SD, standard deviation; STEMI, ST-elevation myocardial infarction; UAP, unstable angina pectoris.

**Table 2:** Medication and procedure in patients with ACS and COPD according to β-blocker use.

Medication	Early use of β-blocker (*n* = 540)	Non-early use of β-blocker (*n* = 544)	*P-*value
Medication within 24 h after admission, *n* (%)			
Aspirin	491 (90.9)	441 (81.1)	<0.001
P2Y12 inhibitor^a^	414 (76.7)	392 (72.1)	0.082
Aldosterone antagonist	118 (21.9)	109 (20.0)	0.463
ACEI/ARB	331 (61.3)	177 (32.5)	<0.001
Statin	517 (95.7)	496 (91.2)	0.002
Procedure			
PCI (%)	306 (56.7)	287 (52.8)	0.196
CABG (%)	3 (0.6)	5 (0.9)	0.484

ACEI, angiotensin-converting enzyme inhibitors; ARB, angiotensin receptor blockers; CABG, coronary artery bypass graft; PCI, percutaneous coronary intervention.

^a^ P2Y12 inhibitor, including clopidogrel and prasugrel.

### 3.2 Association between early β-blocker therapy and in-hospital outcomes

**Figure 2:**
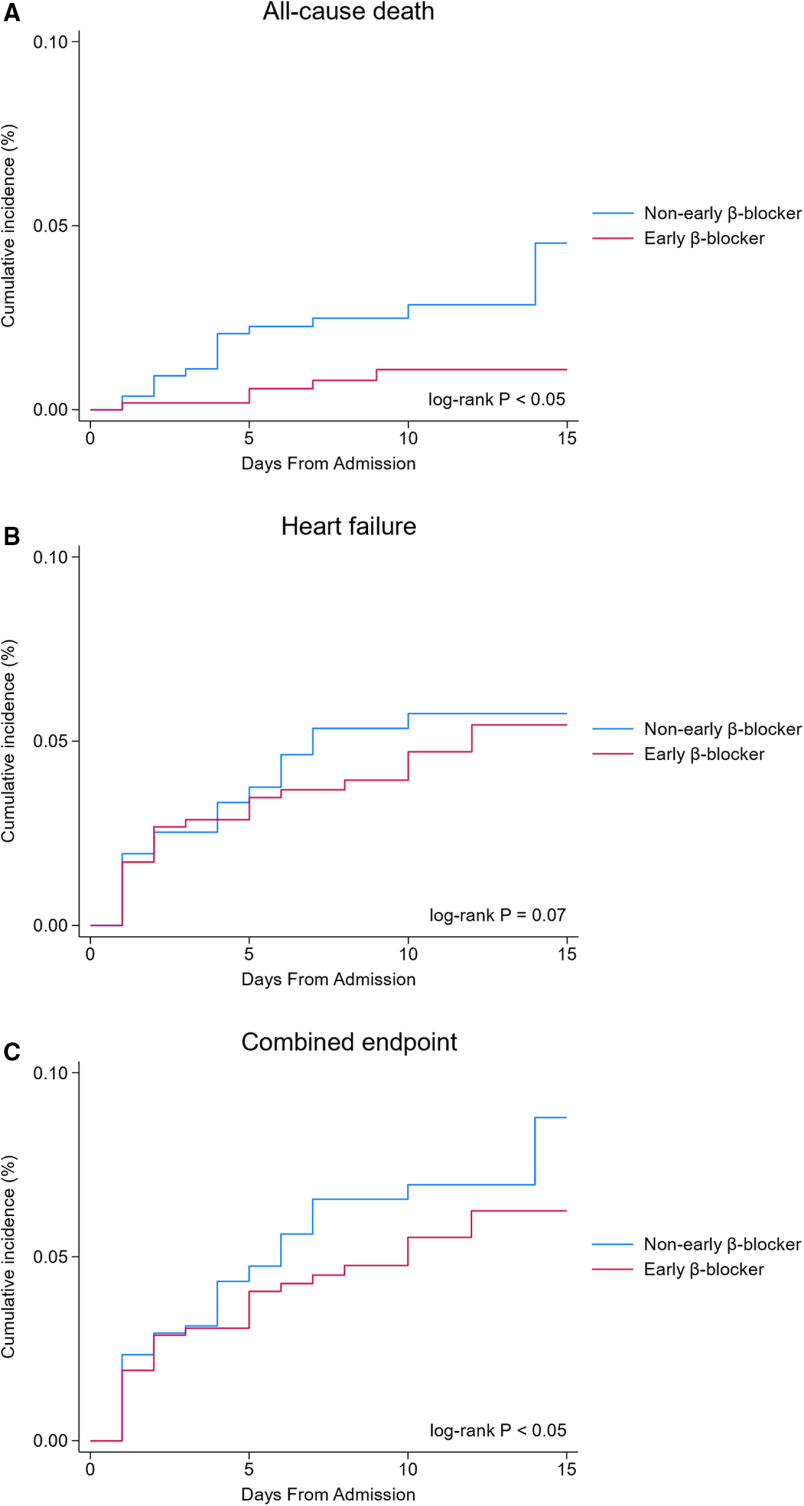
In-hospital clinical outcomes (unadjusted analysis). Early β-blocker treatment group (blue line) vs. non-early β-blocker treatment group (red line).

The Kaplan–Meier survival analysis showed that the incidence of the all-cause death (0.9 vs. 2.9%, *P* < 0.05, Figure 2A) and combined endpoint event (8.2 vs. 12.0%, *P* < 0.05, Figure 2C) were significantly lower in the early β-blocker treatment group compared to the non-early β-blocker treatment group.

The association between early β-blocker therapy and the risk of in-hospital outcomes was analyzed using IPTW-weighted Cox regression analysis. After the IPTW, the absolute standard deviations of the baseline characteristics of the patients in the two groups were all <10%. The characteristics of the two groups after IPTW are shown in Supplementary Material Table S1. The post-IPTW results indicated that the early β-blocker treatment group had a significantly lower risk of in-hospital all-cause mortality (OR = 0.332, 95% CI: 0.119–0.923, *P* = 0.035), heart failure (OR = 0.625, 95% CI: 0.414–0.943, *P* = 0.025), and combined endpoint event (OR = 0.616, 95% CI: 0.418–0.908, *P* = 0.014) than those of the non-early β-blocker treatment group (Table 3).

**Table 3:** Associations between early oral β-blocker therapy and in-hospital outcomes.

** **	Study group		*P*-value	Unadjusted			After IPTW		
	Early use of β-blocker (*n* = 540)	Non-early use of β-blocker (*n* = 544)		OR	95% CI	*P*-value	OR	95%CI	*P*-value
All-cause death	5 (0.9)	16 (2.9)	0.016	0.307	0.112–0.838	0.021	0.332	0.119–0.923	0.035
Heart failure	40 (7.4)	57 (10.5)	0.077	0.695	0.464–1.041	0.077	0.625	0.414–0.943	0.025
Combined endpoint	44 (8.2)	65 (12.0)	0.038	0.668	0.456–0.980	0.039	0.616	0.418–0.908	0.014

Logistic regression was performed to estimate the propensity score, adjusting for the following variables: age, sex, current smoker, previous disease history (MI, PCI, ischemic stroke), diabetes mellitus, hypertension, Killip class, type of ACS, PCI, pre-hospital medication (β-blocker, statin, ACEI/ARB, aspirin), and medication within 24 h after admission (DAPT, P2Y12 inhibitor, aldosterone antagonist, ACEI/ARB, statin). IPTW was calculated by each individual based on his or her propensity score (PS).

### 3.3 Subgroup analysis

**Figure 3:**
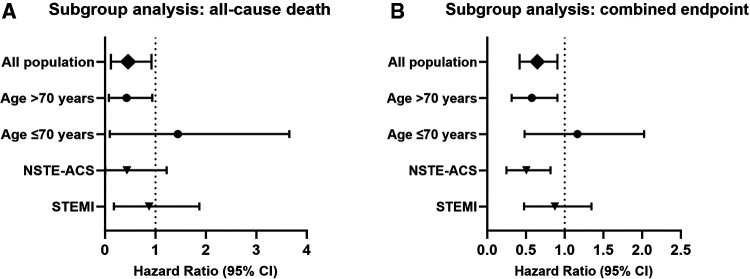
Subgroup analysis (adjusted with IPTW). Hazard ratio and confidence intervals for all-cause death (**A**) and combined endpoint (**B**) in the early β-blocker treatment group compared to the non-early β-blocker treatment group. IPTW, inverse probability of treatment weighting.

Subgroup analyses are shown in Figure 3. Patients older than 70 years with COPD after ACS had a hazard ratio of 0.268 (95% CI 0.077–0.938) for in-hospital all-cause mortality, while those aged 70 years or less had a hazard ratio of 0.586 (95% CI 0.094–3.654) (Figure 3A), suggesting that older patients may benefit more from early β-blocker use. Patients with non-ST-segment elevated ACS had a hazard ratio of 0.073 (95% CI 0.004–1.223) for in-hospital all-cause mortality, while those presented as ST-segment elevated ACS had a hazard ratio of 0.579 (95% CI 0.179–1.872).

The hazard ratio for the combined endpoint events was 0.504 (95% CI 0.316–0.805) in patients older than 70 years (Figure 3B). Patients aged 70 years or younger had a hazard ratio of 0.988 (95% CI 0.482–2.026). Patients with non-ST-segment elevated ACS had a hazard ratio of 0.451 (95% CI 0.249–0.817) for combined endpoint events, indicating that patients with non-ST-segment elevated ACS may also benefit from early β-blocker use. Patients with ST-segment elevated ACS had a hazard ratio of 0.801 (95% CI 0.477–1.346).

## 4 Discussion

β-Blocker therapy was underused in Chinese patients with COPD after ACS. Early oral β-blocker therapy was administered to only 49.8% of patients at admission in this specific population cohort. We found that early oral β-blocker therapy was independently associated with a lower incidence of in-hospital outcomes (heart failure and all-cause death) in patients with ACS and COPD. Our results suggested that patients with COPD after ACS initiated with early oral β-blocker treatment had a lower incidence of in-hospital heart failure and all-cause death compared to those who were not prescribed with β-blockers.

The underuse of the β-blockers in patients with COPD after ACS is a worldwide phenomenon. Our findings in China are consistent with those of the previous studies in other countries (10–13). However, the frequency of β-blocker prescriptions in the present study was much lower than that reported in studies from Western countries. For example, a Swedish nationwide study reported an 84.1% β-blocker prescription rate at discharged COPD patients after MI (13). A study based in the US indicated a marked increase in the use of β-blockers from 64% in 1997 to 93% in 2007 in patients with COPD who developed AMI (11). Meanwhile, another study from the US found β-blocker prescription rates of 65.6% at admission and 77.2% at discharge in ACS patients with a reactive airway disease history (12). Compared with the practices in Western countries, β-blockers in COPD patients following ACS are underused in China. Our study showed that less than one-half of the patients with ACS and COPD were prescribed with β-blocker upon admission. The present study also found that the group using β-blocker had a higher prevalence of hypertension. At the same time, this group was more often prescribed aspirin, ACEI/ARB, and statin. Overall, patients with COPD and ACS who simultaneously had hypertension were more likely to take β-blockers. This might be because β-blockers are commonly used for patients with hypertension in China.

We found that early use of β-blockers after ACS was associated with better in-hospital outcomes, benefiting from a reduction in the incidence of death and heart failure. This correlation was more pronounced in elder patients. Heart failure is common in patients with AMI and is the strongest predictor of death (14). A previous study indicated that respiratory disease was independently related to ischemia heart disease and heart failure (15). COPD was found to be an independent predictor of heart failure in patients with AMI (16). There is evidence that β-blockers do not adversely affect the lung function of patients with COPD (17, 18). Thus, we should be more determined and certain in prescribing β-blockers in COPD patients following ACS as this population could benefit more from β-blocker use.

Several limitations should be acknowledged. This is a multicenter, observational retrospective analysis; therefore, there might be a certain degree of residual confounding owing to the nature of the study. Furthermore, a wide range of physicians were involved in diagnosing COPD patients; thus, the diagnostic criteria might be heterogeneous. The CCC program collected information on whether patients received oral β-blocker therapy within 24 h of admission, but did not record the duration and daily dose of β-blocker use during the hospitalization. Hence, we were unable to further analyze the relationship between the dose-dependent effect of β-blockers and in-hospital outcomes. The effects of different treatment patterns of early β-blocker usage on in-hospital outcomes could be further evaluated in future studies if the daily β-blocker dosage data during hospitalization were available.

## 5 Conclusion

β-Blockers are underused in Chinese patients with COPD after ACS. The early use of β-blockers in patients with COPD after ACS, which is defined as β-blocker administration within 24 h post-admission, was associated with a lower incidence of in-hospital all-cause death and heart failure when compared to non-early use of a β-blocker. The results suggest that patients with ACS and COPD may benefit from the early use of β-blockers.

## Data Availability

The original contributions presented in the study are included in the article/Supplementary Material, further inquiries can be directed to the corresponding author.
