# Supplementary material for: Early β-blocker use and in-hospital outcomes in patients with chronic obstructive pulmonary disease hospitalized with acute coronary syndrome: findings from the CCC-ACS project
Source: Front Cardiovasc Med. 2024 Jul 11;11:1385943. doi: 10.3389/fcvm.2024.1385943 (PMC11269115; doi:10.3389/fcvm.2024.1385943)
Supplement: Supplementary file 1 [file Datasheet1.pdf]

Table S1. Baseline characteristics before and after inverse probability weighting.

	Before IPTW			After IPTW		
	Early use of β-blocker (n=540)	Non-early use of β-blocker (n=544)	*P* value	Early use of β-blocker	Non-early use of β-blocker	*P* value
Age, year, mean (SD)	72.6 ± 9.2	73.0 ± 9.7	0.552	72.9 ± 9.4	72.5 ± 9.7	0.516
Male, n (%)	419 (77.6)	416 (76.5)	0.661	76.9	79.5	0.292
BMI, kg/m^2, mean (SD)	23.5 ± 3.6	23.4 ± 4.1	0.867	23.3 ± 3.4	23.6 ± 4.1	0.221
Current smoker, n (%)	214 (39.6)	226 (41.5)	0.521	39.7	35.3	0.134
HBP, n (%)	396 (73.3)	365 (67.1)	0.025	69.5	71.6	0.442
DM, n (%)	191 (35.4)	193 (35.5)	0.970	33.3	33.5	0.938
Dyslipidaemia, n (%)	59 (10.9)	52 (9.6)	0.458	11.4	9.5	0.306
STEMI at presentation, n (%)	248 (45.9)	261 (48.0)	0.499	47.3	49.7	0.422
Prior comorbidity, n (%)						
Heart failure	44 (8.1)	51 (9.4)	0.475	6.9	9.4	0.126
Myocardial infarction	66 (12.2)	50 (9.2)	0.107	10.3	8.4	0.292
Stroke	77 (14.3)	71 (13.1)	0.563	13.9	12.0	0.347
Peripheral arterial disease	27 (5.0)	23 (4.2)	0.545	4.3	4.1	0.914

Table S1. Baseline characteristics before and after inverse probability weighting.

	Before IPTW			After IPTW		
	Early use of β-blocker (n=540)	Non-early use of β-blocker (n=544)	*P* value	Early use of β-blocker	Non-early use of β-blocker	*P* value
Atrial fribrillation	36 (6.7)	30 (5.5)	0.428	5.7	4.9	0.548
History of myocardial revascularization, n (%)						
PCI	63 (11.7)	49 (9.0)	0.150	10.4	8.2	0.194
CABG	4 (0.7)	2 (0.4)	0.408	0.7	0.9	0.784
Blood pressure, mean (SD), mmHg						
SBP	132.4 ± 21.7	132.2 ± 21.8	0.857	131.5 ± 21.2	134.2 ± 22.1	0.034
DBP	77.8 ± 13.6	76.5 ± 12.8	0.112	77.5 ± 13.2	77.5 ± 12.6	0.954
Heart rate, beats/min, mean (SD)	80.7 ± 14.8	79.7 ± 17.6	0.331	81.2 ± 14.4	80.4 ± 17.7	0.399
Killip class Ⅱ - Ⅲ, n (%)	211 (39.1)	258 (47.4)	0.006	42.9	44.3	0.636
eGFR, mean (SD), ml/min/1.73m**2**	76.2 ± 21.2	75.3 ± 23.4	0.552	76.6 ± 21.8	76.6 ± 22.9	0.956
Renal insufficiency, n (%)	113 (20.9)	132 (24.3)	0.176	20.6	21.7	0.664

Table S1. Baseline characteristics before and after inverse probability weighting.

	Before IPTW			After IPTW		
	Early use of β-blocker (n=540)	Non-early use of β-blocker (n=544)	*P* value	Early use of β-blocker	Non-early use of β-blocker	*P* value
Serum creatinine level, mean (SD), mg/dL	1.0 ± 0.6	1.0 ± 0.5	0.788	1.0 ± 0.6	1.0 ± 0.4	0.968
Hemoglobin, g/L, mean (SD)	132.7 ± 20.4	131.1 ± 20.4	0.221	131.0 ± 21.5	133.5 ± 21.0	0.058
LVEF, mean (SD), %	55.4 ± 9.9	55.1 ± 9.9	0.651	55.3 ± 9.8	55.1 ± 9.9	0.784

Abbreviations are as in Table 1.
